# Changes in cortical thickness: yet another indication of supraspinal adaptations in degenerative cervical myelopathy

**DOI:** 10.1093/braincomms/fcae322

**Published:** 2024-09-20

**Authors:** Aria Nouri, Granit Molliqaj, Karl Schaller, Enrico Tessitore

**Affiliations:** Division of Neurosurgery, Geneva University Hospitals, Geneva 1205, Switzerland; Division of Neurosurgery, Geneva University Hospitals, Geneva 1205, Switzerland; Division of Neurosurgery, Geneva University Hospitals, Geneva 1205, Switzerland; Division of Neurosurgery, Geneva University Hospitals, Geneva 1205, Switzerland

## Abstract

This scientific commentary refers to ‘Patterns of cortical thickness alterations in degenerative cervical myelopathy: associations with dexterity and gait dysfunctions’, by Muhammad *et al*. (https://doi.org/10.1093/braincomms/fcae279).

**This scientific commentary refers to ‘Patterns of cortical thickness alterations in degenerative cervical myelopathy: associations with dexterity and gait dysfunctions’, by Muhammad *et al*. (https://doi.org/10.1093/braincomms/fcae279).**

Degenerative cervical myelopathy (DCM) is becoming increasingly recognized as the most common form of spinal cord injury.^1^ While it has become clear over the last decade that surgery is indicated for patients with moderate or severe impairment, there remains less evidence with regard to the management of mildly impaired patients and ‘asymptomatic spinal cord compression’.^2,3^ Furthermore, there is a well-known clinical recognition of discordance between the severity of spinal cord injury on MRI and the degree of clinical severity; wherein some patients with only minor imaging evidence can present severely from a clinical perspective, while others can present with very significant imaging evidence but can be practically asymptomatic.^4^

These clinically relevant problems may be interrogated in better detail with the understanding of ‘supraspinal’ adaptations, referring to changes that occur in the brain and brain stem, which can occur in patients with DCM. This is because such changes may be able to better stratify disease, provide more quantitative insights into the natural history of DCM and help predict outcomes better than current tools.^5,6^ Although the concept of ‘supraspinal’ changes in spinal cord injury was proposed some time ago,^7^ there has been a significant recent increase of evidence of these adaptations in patients with DCM.^8^

In their article, Muhammad *et al.*^9^ show that cortical thickness is diminished in patients with DCM in multiple cortical regions, including not only the motor and sensory cortex but also the visual cortex, the transverse temporal cortex and pars triangularis. This finding supports similar reported research showing diverse cortical and non-cortical volume loss in DCM in the pre- and post-central gyrus, cerebellum, deep brain structures and the brain stem.^10,11^ However, one must wonder, if cortical volume reductions are present in so many regions, how can this be used clinically, other than to interpret this as wide-ranging atrophy? Additionally, does DCM only affect motor function and sensation, or can these wide-spread cortical changes have an effect on other important functions?

Indeed, while the authors’ finding of cortical volume loss is notable, it is important to consider that other mechanisms of ‘supraspinal’ brain changes have been reported and must be considered. These include functional connectivity and neuroplasticity. For example, while visual cortex volume may be diminished in size, others have demonstrated that bilateral insular cortices, and the supplementary motor area (SMA) exhibited greater functional connectivity with the visual network—this resulted in the hypothesis that DCM patients impart greater reliance on visual inputs as a compensatory mechanism for their gait problems.^12^ One could interpret this as no direct volume loss, but perhaps a re-organization. Following this line of thinking, it has also been suggested that DCM patients present with a variable ‘corticospinal reserve capacity’, and that cortical loss in the motor area can be potentially compensated by neuroplasticity changes in adjacent regions such as the SMA.^13^ Ultimately, these findings suggest that cortical thinning or volume loss cannot be considered by itself and should be considered in conjunction with these other relevant adaptive mechanisms.

One of the main constraints of research into ‘supraspinal’ adaptive changes in DCM remains the limited patient size of studies; however, this is largely understandable as this topic remains in the early phases of investigation. Furthermore, as the authors have correctly pointed out, clinical measures that are currently used to assess the clinical manifestations of DCM, such as the mJOA, remain somewhat crude, and therefore the authors have attempted to use more specific measures such as dexterity and gait analysis to provide more objective assessments. While this is encouraging, using specific clinical measures needs to be balanced with the clinical usability and availability of such tools in everyday practice. Finally, one must also consider that DCM is a heterogenous disease with very different clinical manifestations of spinal cord injury. For example, some have predominant gait abnormalities, hand weakness or loss of dexterity, and yet others present with concomitant radicular or neuropathic pain. Therefore, one cannot expect these patients to present with equivalent adaptive changes in the brain. This problem of heterogeneity does not only apply to research on adaptive brain changes but has also been appreciated in the assessment of clinical outcomes in DCM in general. Indeed, this has recently spurred a proposal of clustering DCM patient into different clinical phenotypes.^14^

Overall, Muhammad *et al.*^9^ provide an interesting addition to the mounting literature supporting the role of cerebral re-organization as a diagnostic tool in DCM. Studies such as theirs, in conjunction with research on functional adaptations and neuroplasticity, will provide important insights into the pathophysiology of DCM, and eventually may provide an alternative avenue for assessing disease severity. Furthermore, the combined assessment of brain volumetric changes, connectivity and neuroplasticity will allow for the evaluation of patient-to-patient differences in their adaptive capacity. This will ultimately allow for more precise treatment strategies that will address some of the ongoing knowledge gaps and may open the door to completely new treatment paradigms.

## Data Availability

As this is a commentary, there is no relevant data associated with this manuscript.
